# COVID-19 in Saudi Arabia: An Overview

**DOI:** 10.3389/fpubh.2021.736942

**Published:** 2022-02-02

**Authors:** Asharaf Abdul Salam, Rshood M. Al-Khraif, Ibrahim Elsegaey

**Affiliations:** Center for Population Studies, King Saud University, Riyadh, Saudi Arabia

**Keywords:** epidemiology, proportions to world, percentage of population, local distributions, administrative areas

## Abstract

**Background:**

Saudi Arabia, a prominent Arabian country, has 35. 3 million persons living in 2.2 million square kilometers, undergone serious threats recently due to the COVID-19 pandemic. With the built-in infrastructure and disciplined lifestyle, the country could address this pandemic.

**Aims:**

This analysis of COVID-19 cases in Saudi Arabia attempts to assess the situation, explore its global percentage share, percentage of population affected, and local distribution from the beginning of infection until recently, tracing historical developments and changes.

**Data and Methods:**

This analysis made use of data released by the Ministry of Health on a daily basis for a number of parameters. They are compiled on an excel sheet on a daily basis: the dataset has undergone rigorous analysis along with the trends and patterns; proportion to the world statistics and geographic distribution.

**Results:**

COVID-19 spread rapidly in the country with periodic variations, during June-August, 2020. But, recoveries accelerated in the period, thus bridging the gap of increasing infections. In comparison with the world statistics, the country proportions are lower, while the percentage of population affected is similar. It appears that the intensity varied across all 13 administrative areas.

**Conclusion:**

COVID-19 transmission since March 2020 is considered to be widespread, creating excess burden on the public health system, delineated into stages (early infection, rapid spread, declining, stabilizing, and second wave). Control measures are set, stage-wise, without impinging upon normal life but to ensure that the proportion of globally affected persons is lesser than the population share: credit goes to the Ministry of Health. Area-wise spread depends largely on population density and development infrastructure dimensions. Ultimately, the disciplined life in compliance with law and order paved the way for effective program implementation and epidemic control.

## Introduction

The COVID-19 pandemic spread rapidly across the world, including Saudi Arabia, which led to a severe health emergency (1–5). There are many facets of spread in the country with variations across populations, geographies, and families. Despite all efforts of the government health system and responsible residents, the pandemic spread faster but was controlled through intervention strategies of the Ministry of Health such as digital health, social distancing, suspending gatherings, temporary closures, and imposing curfew at commercial and service utilities (6–8). Simultaneously, interventions were phased out at the national level considering infections, mortality, and recovery with geographic importance (9–14).

The epidemic period combined with health emergencies created tensions in family units, especially under poor conditions of infrastructure and crowded living arrangements due to restrictions on family and social life, interpersonal contact, and affective gestures adhering to the strict discipline of social distancing and face masks (5). This lead to containment: quarantine, lockdowns, and curfews in turn creating conflicts, tensions, and violence manifesting upon contact with infected persons (1, 15, 16). Consequently, it leads to an overall breakdown of the individual, family, and society with enormous changes and unparalleled consequences-financial and medical (17, 18). Moreover, there are economic, social, and community impacts (10, 19) despite the assessments and investigations from the medical perspective and daily data release from the Ministry of Health.

Saudi Arabia, a large country in terms of geographic area, is divided into five planning regions, 13 administrative areas, and 118 governorates. It borders five Arabian Gulf countries and a few other Arab countries, and accommodates a combined native and foreign population of 35.3 million across 2.2 million square kilometers. This predominantly urban country built residential, commercial, educational, medical, and other infrastructure to encourage community living, which expedites the possibility of faster infection (8, 20, 21). Floating population, despite the efforts of containing, isolating, social distancing, and closing, causes the spread of COVID-19 in a new form of Middle East Respiratory Syndrome Coronavirus (MERS-CoV) that plagued the Middle East, although a majority of cases were reported in Saudi Arabia (3, 22–27). These reports of this worldwide pandemic in Saudi Arabia are attributed to population size, resisted through strategic interventions and mitigation measures characterized by swift community action and hospital preparedness (3, 10, 26, 28–31). This aligns with vision 2030 that positions the country as a business and tourism hub (8, 32).

Against this backdrop, this research aims at an epidemiological analysis of COVID-19 cases reported on a daily basis to highlight changes, patterns, and trends over a period from March 21, 2020 to November 22, 2021. With the limitations of the national-level data available for analysis, this research elucidates the path of COVID-19 infection in the country, from its very beginning until recently. Such an elaboration, which has not yet been attempted, might enlighten researchers, policymakers, and practitioners to track historically and to learn lessons from a successfully implemented infection control program. Not only does this elaboration exposes the Saudi Arabian experience to global readers, but also it gives data and insights to the rest of the world, especially the Arab countries.

## Data and Methods

This empirical study adopting ex-post facto approach is based solely on daily status reports of the Ministry of Health of Saudi Arabia published since March 21, 2020. Calculations were done to determine:

a. daily changes in infection
b. daily differences between reported and recovered cases
c. daily changes in active cases and critical cases
d. case fatality rate (deaths until date per 1,000 infections until date)
e. new and active cases, recovery and mortality as a percentage of global
f. infections, recoveries, and deaths per 100,000 population

Data published contained city-wide data till November 28 2020; thereafter it was by the 13 administrative areas till September 25, 2021; and at national level totals there. There were reports of global figures too till September 25, 2021. It was owing to the substantial decline in infection that these changes in the data structure are enacted by the Ministry of Health. Furthermore, adopting population size (35,575,968 for October-November; 35,484,062 for September; 35,439,591 for August and 34,543,959 for others as cited on www.worldometers.info) as a denominator, indicators such as (a) daily reported cases, (b) total cases and total recoveries, (c) total deaths, (d) critical cases, (e) active cases, and (f) vaccinations are calculated for a base population of 100,000.

These analyses were based solely (as a source of data) on daily reports of COVID-19 cases published on the Website and through social media platforms (Facebook) by the Ministry of Health, Saudi Arabia. These reports from March 21, 2020 to November 22, 2021 are compiled on an Excel worksheet for consolidated analyses and illustrations.

## Results

Results of this analysis are presented under various headings: epidemiology, proportion to world statistics, percent of the population affected, and spread by administrative area.

### Epidemiology

**Figure 1:**
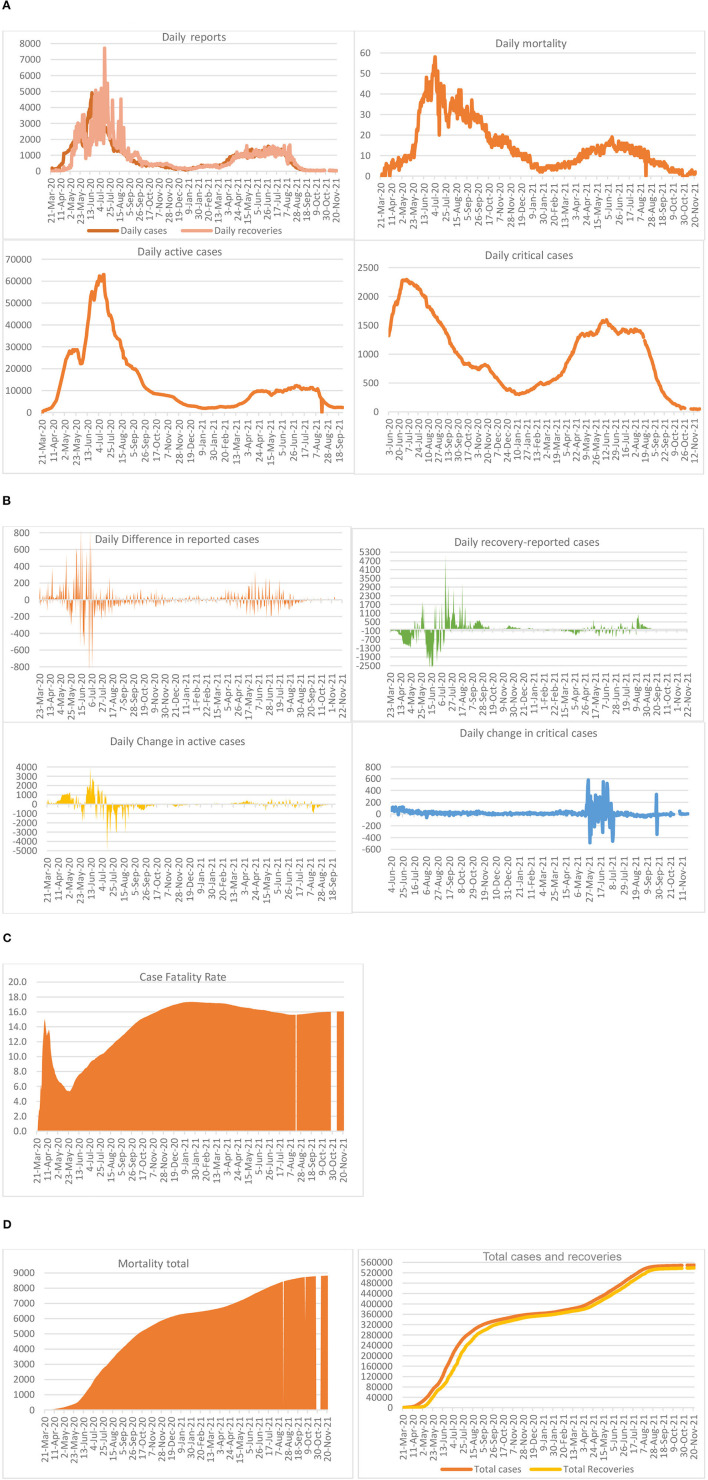
Epidemiology of COVID-19 in Saudi Arabia. **(A)** Daily reports, **(B)** differences in daily reports, **(C)** case fatality, and **(D)** outcomes in total.

A total of 392 cases of COVID-19 recorded on March 21, 2020, increased to 549,518 on November 22, 2021 showing a rapid spread in a population of 35.3 million; unexpected and un-afforded to the public health system. Fortunately, there were reductions in reported infection per day from 4,757 on June 18, 2020 to 220 on November 28, 2020; 328 on February 28, 2021; 1,161 on June 7, 2021 and 39 on November 22, 2021 (Figure 1). Along with this decrease in the infection was the mortality due to the pandemic, which declined with the infections but at a lesser proportion. From a single death reported on March 24, 2020, daily deaths increased to 58 on July 5, 2020. The death toll per day remained high at 13 on November 28, 2020; 6 on February 28, 2021; and 15 on June 7; 2021 but declined to 2 on November 22, 2021. Together with these indicators are the daily recoveries, which exceeded new cases since May 12, 2020, but with minor fluctuations. It appears that the gap between active cases and recovery existed during June - July 2020 started to decline slowly since October 2020, and rapidly thereafter.

Two of the important indicators are active cases and critical cases: the former, as reported, increased from 22,444 on June 3, 2020, to 63,026 on July 13, 2020, but declined sharply thereafter. While the increase from June 3 to June 19 was sharp, it slowed down thereafter, reaching 45,157 on July 22, 2020; 23,687 on August 23, 2020; and 8,487 on October 19, 2020; 1,894 on January 16, 2021; and 2,296 on September 25, 2021. On November 28, 2020, there were 5,018 active cases (1.4% of infected cases, leaving 1.6% deaths), which declined to 2,584 by February 28, 2021; 2,581 on March 4, 2021 but thereafter increased to 9,376 on June 7, 2021. While the rapid increase in active cases noted during March 21-June 12, 2020 could be considered as part of the first phase, those noted in 2021 could be explained as part of the second phase. The total number of infections and recoveries has been varied but bridged the gap since October 2020. Moreover, the gap widened and shortened depending upon the daily reports. On the contrary, dwindling changes in the critical cases were recorded on a daily basis until the end of January, and started to climb up thereafter. For example, on June 3, 2020 there were 1,321 critical cases that increased to a peak of 2,295 on July 4, 2020, decreased to 352 on January 30, 2021 and thereafter increased to 1,579 on June 7, 2021. The same started falling slowly, thereafter, reaching a figure of 52 on November 22, 2021.

Increases in the daily number of infected cases were in multiples of hundred during the early days of COVID-19. For example, on March 24, 2020, the number of new cases was increased by 154. But on July 3, 2020 the highest daily increase was reported as 810, which reduced from August onwards. On the contrary, there were reductions too, for example, on April 8, 2020, there was a reduction of 135 cases. Along with the spread of infections, mitigation also took place resulting in recoveries from the episodes. As of February 28, 2021, 368,305 cases have been recovered out of a total number of infections of 377,383 cases reported, representing a 97.6% recovery rate, leaving 6,494 (1.7%) deaths, which shows a prevalence of 0.7%. During the early days, that is, March and April 2020, daily recoveries were lower than reported cases, which is the reason for a huge increase showing negative recovery-reported case statistics. For example, on March 24, 2020, this difference was 196, with 9 recoveries out of 205 cases. On May 4, 2020, this figure of recovery-reported cases reached −1,303 and on June 12, 2020 it reached a peak of 2,911 cases, adding up to active cases. On the other hand, there were recoveries exceeding new cases from May 12, 2020 onwards but were zigzagging: the highest on July 14, 2020 with a difference of 5,026 cases (7,718 recoveries as against 2,692 new cases).

The case fatality rate, calculated as total deaths to 1,000 total infected cases, reached 16.3 on June 7, 2021. It was recorded as 15.1 on April 6, 2020, which declined to 5.4 by May 23, 2020. However, case fatality was recorded at a high of 17.3 during January 6–February 16, 2021, and 8,826 on 22 November, 2021 depending mostly on the positive cases and deaths reported. The total number of deaths reached 7,471 on June 7, 2021 with a daily mortality of 16. The highest number of deaths of 58 was reported on July 5, 2020.

### Proportion to World Statistics

**Figure 2:**
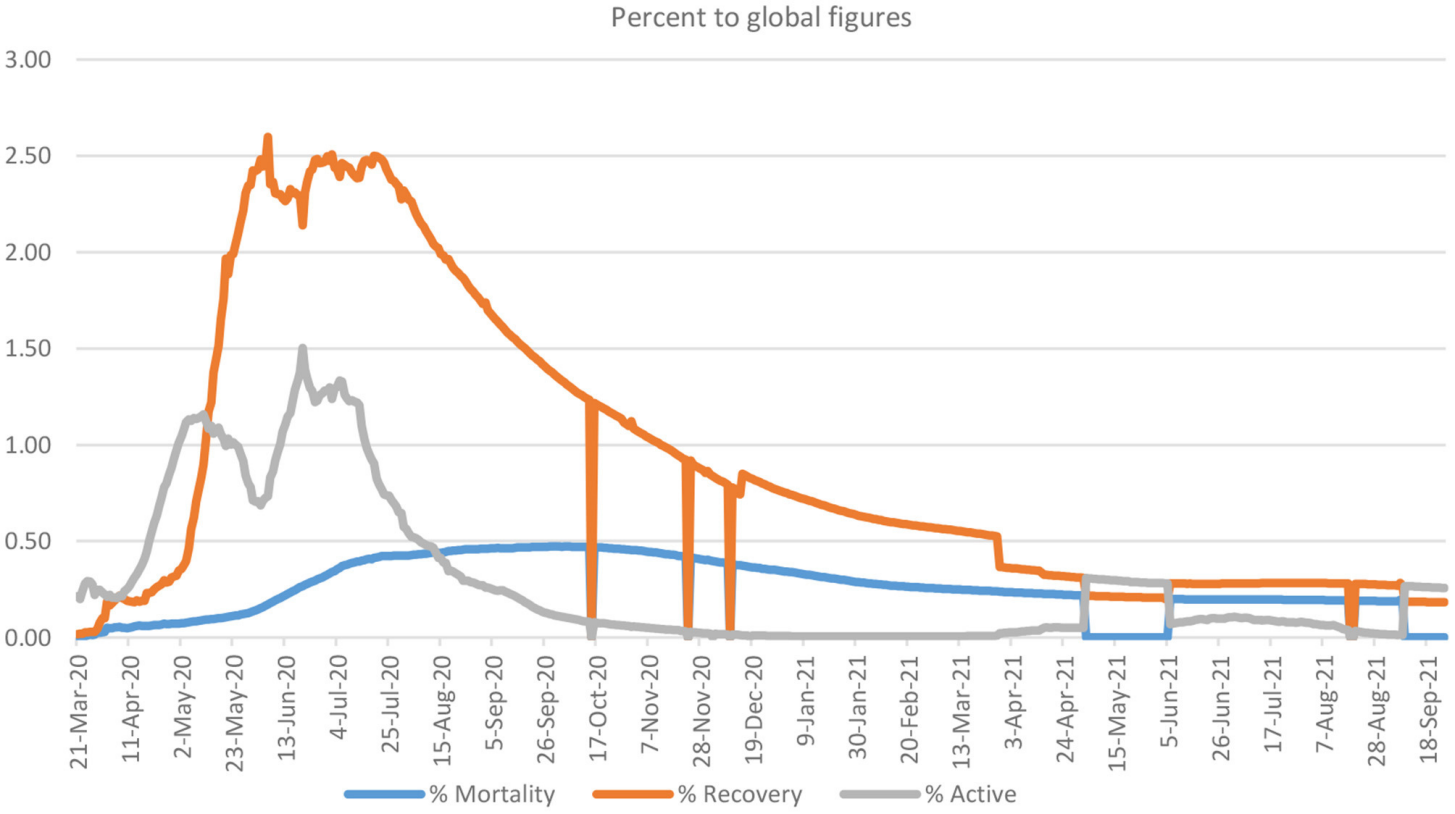
Global comparisons of COVID-19 statistics.

Saudi Arabia, the largest country in the Arabian Gulf and second largest in the Arab World, has an area and population that are both 0.4% of the world. Saudi Arabia started with 0.2% of the world's COVID-19 cases on March 21, 2020, which increased to 1.5% by June 20, 2020, but declined to 0.02% by November 28, 2020, 0.01% by February 28, 2021, and even less than that thereafter, following various phases of intervention over that period (Figure 2). Recoveries were low from March 21 to May 4, 2020, sharply increased during May 5–20, fluctuating with high recoveries until August 4, 2020, and lower recoveries thereafter. Still, the percentage of patients recovered fluctuated between 0.02 and 2.5% of global figures.

### Share of the Population Affected

**Figure 3:**
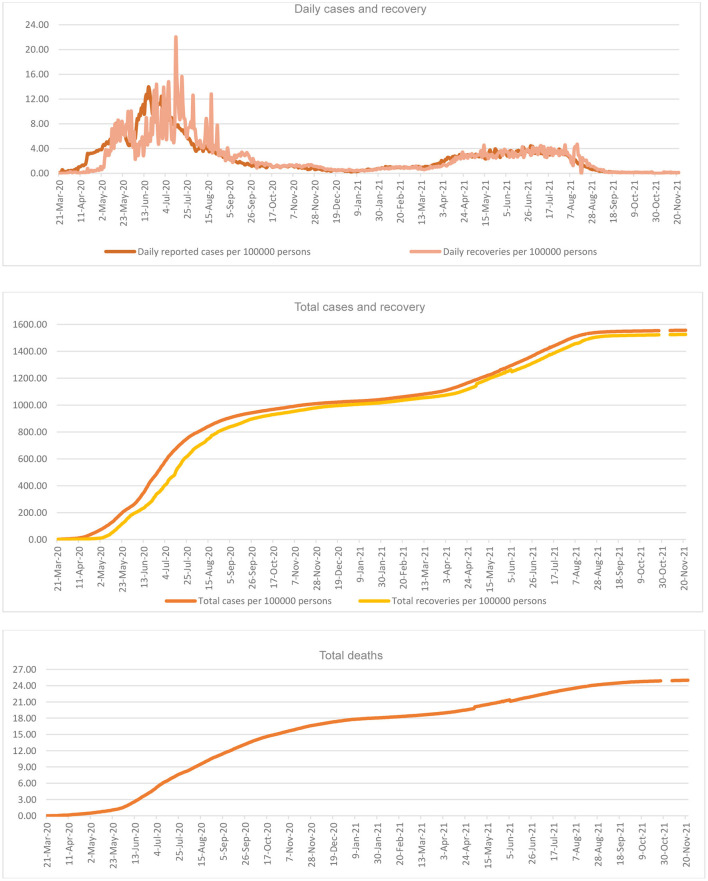
COVID-19 Infections in per 100,000 persons in Saudi Arabia.

There are a total of 549,518 infected cases, as of November 22, 2021 (1,556 per 100,000 persons); higher rates of infection but almost all recovered (538,640; 1,526 per 100,000 persons). Thus, having a very narrow gap between infected and recovered persons (Figure 3). It is the mortality from COVID-19, that receives greater concern in the country, especially due to its higher number per 100,000 persons. Daily reports of cases also show reductions but with intermittent increases. While applying the total population as the denominator, daily infection of COVID-19, as plotted, reached its peak point of 14 per 100,000 persons on June 17, 2020 on the day of the highest number of infections of 4,919 in the country. This percentage declined sharply thereafter to 1 on February 28, 2021; 3 since April 8, 2021, but was negligible by November 22, 2021. While the daily cases declined to the lowest (0.23 per 100,000 persons) on January 3, 2021, it shoots up thereafter for a short period. Similarly, the total cases had declined to 1,028 on January 3, 1,043 on February 28, 1,182 on April 30, and 1,299 on June 7, per 100,000 persons: total recoveries were 1,018, 1,058, 1,151, and 1,269; and total deaths were 18, 19, 20, and 21 per 100,000 persons, respectively.

### Spread by Administrative Areas

**Figure 4:**
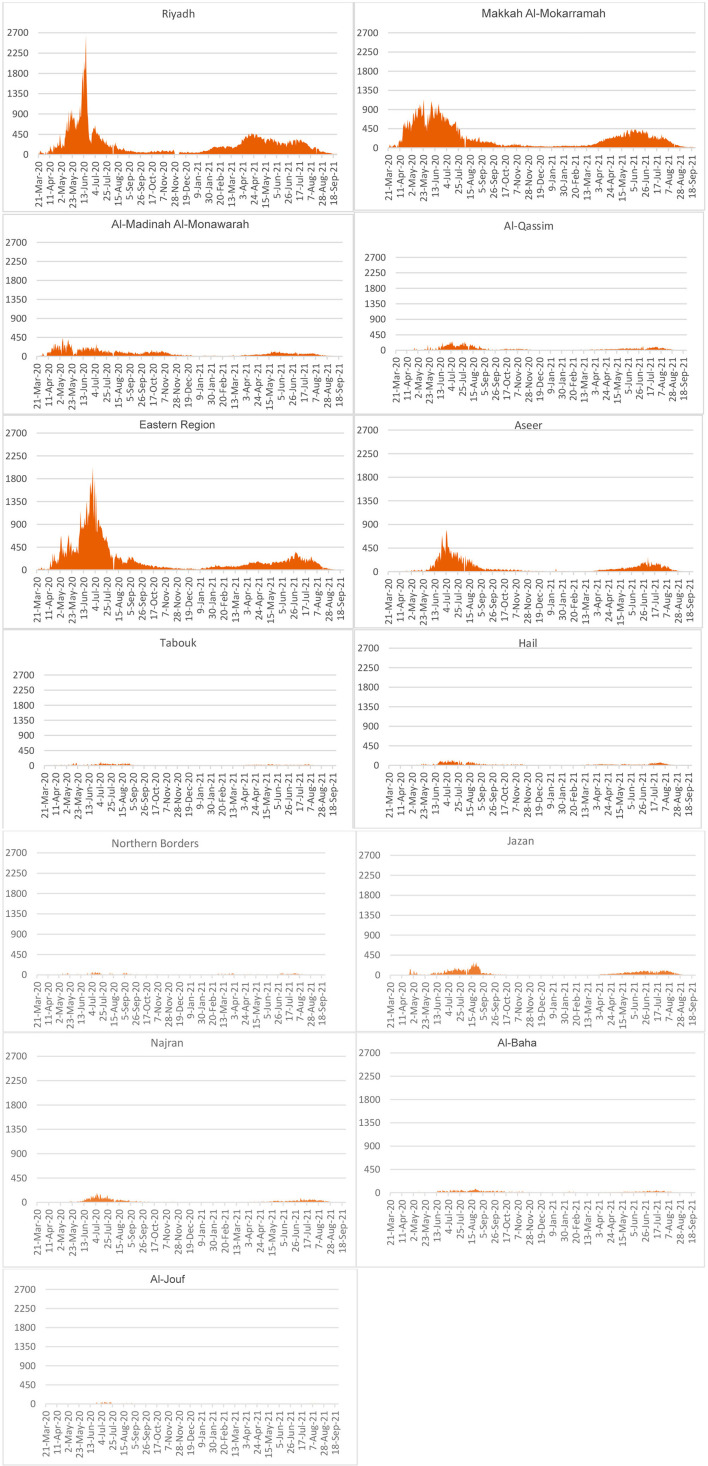
Spatial distribution of infections reported daily.

Some of the administrative areas, especially major commercial, educational, residential, and developmental zones, reportedly have a higher number of COVID-19 cases (Figure 4). In Riyadh, there was an upsurge of cases during May-July, 2020 but this has come down since August 2020, reducing to a low by January 2021 but with a slight increase thereafter. Following Riyadh are the Eastern Region and Makkah Al-Mokarramah administrative areas, having been affected seriously. These three administrative areas have higher population pressure and also the proportion of the population affected (1,586, 23,647, and 1,496 per 100,000 population, respectively) than other administrative areas. However, differences across the administrative areas on population size produce differing proportions of infected. This paper has no scope for such a detailed analysis across the geographical divisions.

## Discussions

COVID-19, the most critical health issue humans have ever experienced over the last decade, vary across countries in intensity raising global issues by creating severe health and socioeconomic concerns, thus precipitating global disruption and emergencies affecting other aspects of life, including travel, material, and financial resources, and psychosocial wellbeing (3, 24–26, 33–35). The fast spread of COVID-19 in Saudi Arabia created panic responses from individuals, families, and social groups to adjust with strategies and control measures, including welfare and relief (6). Fast increases followed by reductions on daily new cases and mortality coupled with recoveries periodically, monthly, explicitly displays enactment and adherence of the various strategies of COVID-19 control, in the country, after September 2020 (4, 33). Such a decreasing trend in active cases continued further until February 2021. While the new cases increased with lesser recoveries were characteristic of March-April, 2020, the situation reversed thereafter bringing recoveries exceeding new cases. Moreover, infrastructure created by the public health system for screening, controlling, containing, and quarantine paved way for swift community action and hospital preparedness thereby minimizing the widespread transmission risk enabling a reduction in further spread (1, 4, 9, 30, 36).

Chronologically, risk assessment was followed by suspension of religious, recreational, sports, and commercial gatherings and thereafter public transport regulations leading to a partial curfew. There were enforced restrictions of inter-regional and international and national movements, local curfews based on daily reports, and national level lockdown. Connections are maintained through e-services. Repatriation of citizens, isolation of districts, remote teaching procedures, and rules carrying reprimands for the violation of control measures were also introduced. The private sector and expatriates were offered financial and welfare support along with special terms during Ramadan. Control measures were lifted slowly, step by step, depending upon the locational volume of spread. Mass testing strategies were initiated and, thereafter, normal living was regained in Saudi Arabia. There were a few other control measures put in place during the second wave too, although the spread was less intense. Slowly, there were reductions in the spread and thereby control measures were removed in the Kingdom. By this time, immunization programs gained momentum and it became mandatory for movement, especially in public places and offices. As an outcome, by September 2021, almost all control measures were withdrawn, observing a noticeably low spread of COVID-19. There exist restrictions on international travel and the entry of non-immunized people into the country. This is based on the lessons gained about the onset of the disease carried to the country by frequent travelers of the Eastern Region.

Mortality, measured as case fatality, was observed to be high, which increased rapidly until January 2021: such higher mortality rates might have resulted from population age distribution, the age of infected persons, life expectancy, comorbidity, treatment-seeking behavior, and other risk factors (37). However, an increase in case fatality could be probably attributed to the rapid decline in infected cases, the denominator. Overall mortality levels increased by around 2,000 cases in 2020 compared with that of 2019, which may be specifically attributed to COVID-19. Such hikes in mortality by a specific cause exerts a heavy burden on the public health system.

COVID-19 spread to more than 200 locations in Saudi Arabia, and thus gripped the country for a period, which was addressed through medical and legal intervention. This reduced not only the gap between infection and recovery but also the proportion to global infection, recovery, and mortality. While the global figures continue to increase rapidly, the share of Saudi Arabia declined, which may be credited to the mitigation efforts.

Population size and density are potential sources of COVID-19 infection (38), especially in the Arab culture. This applied to Saudi Arabia, especially the major cities characterized by a modern lifestyle under nuclear families, affecting the traditional family togetherness and cohesiveness. But in comparison to the global scenario, the country's levels are below its population proportions in terms of infections and mortality, which explains the national scenario including implementation of control measures. Such a positive mitigation outcome likely explains the change in lifestyle in line with legal and cultural regulations and COVID-19 control strategies.

There were increases in the overall total cases and mortality, which are attributed to the waves of this epidemic on a global basis. But, threats are limited as revealed by the affected persons as a percentage of the population. Thus, population rates are more meaningful than the absolute numbers for understanding the impact of COVID-19 on societies. These rates show the extent COVID-19 impacts the population regarding distribution, economy, behavior, and cohesiveness, directly and indirectly. These achievements of continued decline are geared by strenuous efforts of healthcare intervention including daily detection tests and vaccination.

Saudi Arabia has gone through highs and lows; based on population size, urban growth, infrastructure in place, and economic sectors. For example, a high spread of disease reported in administrative areas such as Riyadh, Makkah Al-Mokarramah, and the Eastern Region corresponds to this view. The second set of administrative areas are Al-Madinah Al-Monawarrah, Aseer, Al-Qassim, and Jazan. The other areas had few infected cases. These variations across administrative areas could directly relate to urbanization, social and religious festivities, commercial activities, and livelihoods despite effectively implementing various containment measures all over the country (8, 20, 23, 39). Almost all administrative areas passed the peak stage of infection and thereby marked declines with substantial public health measures put in place, which are capable to confront political, monetary, and social difficulties (3, 26, 32). Moreover, a majority of the cases are travelers from other countries in the case of the Eastern Region, and from contacts in the case of Riyadh, Makkah Al-Mokarramah, and Al-Madina Al-Monawarah; apart from medical professionals (40).

## Conclusion

COVID-19 in Saudi Arabia witnessed increases and decreases epidemiologically, in terms of new cases, mortality, active cases, and critical cases, delineating phases of early infections (March-May, 2020), heightened spread (June-July, 2020), fast decline (July-September, 2020), stabilization (September, 2020-April, 2021), second-wave (April-September, 2021), and full control (October 2021 onwards). While country statistics show remarkable control, credit goes to the committed efforts of the Ministry of Health, Saudi Arabia, and disciplined adaptations by the public. Overall, the percentage of population affected is low, comparatively, but should be kept in view for continued efforts to control the virus. Mitigation along with infection control strategies should go hand in hand in a strengthened manner.

Geographically, administrative areas with higher pressures of population migration and socioeconomic development are more affected, especially the major cities such as Riyadh, Jeddah, Makkah, Buraydah, Dammam, and Madina. Finally, it is the disciplined life in compliance with a law and order situation under a government of utmost accountability that enabled the achievement of goals and targets in time.

This research has many limitations, especially those related to data. Still, with this available data on the national scenario, the overall situation is explained for the global audience. It would have been more insightful had there been detailed data on age and sex specificity of infections, recoveries, and mortality. Such details could also be beneficial for analysis across geographic locations.

## Data Availability Statement

The original contributions presented in the study are included in the article/supplementary materials, further inquiries can be directed to the corresponding author.

## Author Contributions

AS: initiation, concept development, data compilation, analysis, and writing. RA-K: advisory in data compilation and methodology, reviewing and revising, and enabling supportive mechanisms. IE: assisting in data compilation, helping in analysis and literature review, and reviewing and suggesting improvements. All authors contributed to the article and approved the submitted version.

## Funding

Deanship of Scientific Research at King Saud University, Riyadh, for its funding of this research through the Research Group No. RGP-329.

## Conflict of Interest

The authors declare that the research was conducted in the absence of any commercial or financial relationships that could be construed as a potential conflict of interest.

## Publisher's Note

All claims expressed in this article are solely those of the authors and do not necessarily represent those of their affiliated organizations, or those of the publisher, the editors and the reviewers. Any product that may be evaluated in this article, or claim that may be made by its manufacturer, is not guaranteed or endorsed by the publisher.
